# Supplementary material for: Stochastic choice drives variability in patch foraging decisions in humans and rats
Source: Commun Psychol. 2026 May 19;4:112. doi: 10.1038/s44271-026-00465-0 (PMC13447090; doi:10.1038/s44271-026-00465-0)
Supplement: Supplementary file 1 [file 44271_2026_465_MOESM1_ESM.pdf]

nature portfolio

Peer Review File

Stochastic choice drives variability in patch foraging decisions in humans and rats

Corresponding Author: Dr Emma Scholey

This file contains all editorial decision letters in order by version, followed by all author rebuttals in order by version.

Version 0:

Decision Letter:

** Please ensure you delete the link to your author homepage in this e-mail if you wish to forward it to your coauthors **

Dear Ms Scholey,

Thank you for your patience during the peer-review process. Your manuscript titled "Stochastic choice drives variability in patch foraging decisions across species" has now been seen by 3 reviewers, and I include their comments at the end of this message. They find your work of interest but raised some important points. We are interested in the possibility of publishing your study in Communications Psychology, but would like to consider your responses to these concerns and assess a revised manuscript before we make a final decision on publication.

We therefore invite you to revise and resubmit your manuscript, along with a point-by-point response to the reviewers. Please highlight all changes in the manuscript text file.

Editorially, we consider that the following concerns need to be carefully addressed. First, the text should be clarified and elaborated to reach a broader audience, following the suggestions of Reviewer#1. Second, Reviewer#2 requests an explanation of the rationale for selecting the chosen models, a detailed description of the model-comparison pipeline, and a more thorough discussion of the theoretical implications of one model outperforming the others. We want these aspects fully addressed. Finally, Reviewer#3 raises substantial concerns about the gain in knowledge the field can derive from the present work; please address this aspect carefully in the revision.

As you revise the manuscript in response to these issues, please also implement all requests in the attached Mandatory Revision Requests document. All requirements listed in this document need to be fully met, or the work will be returned to you for further revisions without peer review. This workflow is in place to increase the likelihood that the paper will be accepted for publication. It reduces the number of rounds of revision (and review) and ensures that the reviewers vet a version of the article that is compliant with journal policies. If you have any questions regarding the required revisions, please contact the journal prior to resubmission to avoid a negative outcome.

We are committed to providing a fair and constructive peer-review process. Please don't hesitate to contact us if you wish to discuss the revision in more detail.

Please submit the following items:

- Revised manuscript
- Point-by-point response to the referees' comments
- Mandatory Revision Requests Table (attached).
- Cover letter (as a separate document)

via this link: Link Redacted .

** This url links to your confidential home page and associated information about manuscripts you may have submitted or are reviewing for us. If you wish to forward this email to co-authors, please delete the link to your homepage first **

We hope to receive your revised paper within 8 weeks; please let us know if you aren't able to submit it within this time so that we can discuss how best to proceed. If we don't hear from you, and the revision process takes significantly longer, we may close your file. In this event, we will still be happy to reconsider your paper at a later date, provided it still presents a significant contribution to the literature at that stage.

We look forward to seeing the revised manuscript and thank you for the opportunity to review your work.

Best regards,

Troby Lui, on behalf of

Eva R. Pool

Troby Lui, PhD
Associate Editor
Communications Psychology

Eva R. Pool, PhD
Editorial Board Member
Communications Psychology
orcid.org/0000-0001-5929-1007

REVIEWER EXPERTISE:
Reviewer #1 Decision making, Foraging, Modeling
Reviewer #2 Decision making, Foraging, Modeling
Reviewer #3 Decision making, Foraging, Modeling

REVIEWER REPORTS:

Reviewer #1 (Remarks to the Author):

This paper tests a stochastic choice model to model variability in patch-leaving decisions in various foraging tasks, across humans and rats.

I really appreciate the idea of comparing across similar experiments and species to understand the fundamentals of decision-making. However, I found the motivations for the paper and the models, presentation of results and the implications to be lacking. Mainly, I am not sure I follow the authors' main claim that environments don't predict choice variability. It is confusing to tell whether certain environments have a greater variability or not, due to the use of two variables (CV and SD), and the model explanations are also hard to follow.

I would suggest the authors to edit substantially to improve the readability of the manuscript, while clarifying why these questions matter, why certain models were used and what the results mean.

Other comments:

1. Abstract could frame the gap or the question better. What is stochastic choice? Why do we care?

2. Figure 1 is quite nice! One suggestion would be to mention what the %mean in (b) in the legend.

3. If the demarcation between early and later trials were to be changed, will the results in Fig 2(b) remain the same? If foragers can learn quickly, then early trials need not be the first half, but rather first 3rd or 4th of all trials. If the authors plot the CV_leave over time, and see whether there is a change point after initial trials, then that would present a clearer picture. This is especially important because theoretically variation in such decisions indicate learning.

4. Relatedly, what is the direction of the CV_leave in terms of overstaying or understaying? could it be that the direction is the meaningful axis that demarcates CV differences between early and late trials -- people could vary from the mean to overstay in early trials, but in later trials they vary more in understaying.

5. Discussion of Fig2a is lacking on Page 6. CV seems to be highest for the incongruent condition of rich environments + low yield, across the 3 tasks, although it is more pronounced in the rat task. This pattern has not been commented upon.

This is crucial because this effect is what the authors are trying to suggest their model speaks against (i.e., environment does not explain variability). In addition, the 3 tasks do have differences. the rat task has a 3-fold difference between the travel times, while the berry task has a very minimal difference.

6. The results from the models are hard to follow. Why did the authors show the CV earlier and then switch to SD in Fig 3 and 4? Around L181, the authors say that variability is independent of the type of patch/environment. This is quite odd because the behavioral results show clearly that rich environment-low yield patches have the highest CV. Plus, in the line 183, the authors say that reward weighting between environments provides a better account. This would mean that sensitivity to the environment matters, right? It is difficult for me to follow the model and the results because of the way they are written, but the inferences are doubtful.

7. Fig 3. Why is the bias, c set to -3.6? and why does it show only negative sweeps of c? If people and rats tend to usually overstay in a patch, then the bias should be > 0, right?

8. L194-98. This is confusing - Fig 2 showed that rich environments+low yield for the rat task had the highest variability, now based on the SD, the authors say that low-yield patches have the least variability.

9. There are many grammatical errors in the manuscript, some below:
72. "were inspired"
73. "types of patches", perhaps
81-82. "the patch effect" and "the environment effect" are written in a way suggesting that these effects by these names have been previously defined, which they have not.
112. I know the hypothesis is stated in the previous line but because of the subsection change and the figure, it doesn't stick. Would help to reiterate.
145. "depend on". the sentence reads odd.
171. "all model variants" or "all models"

Reviewer #2 (Remarks to the Author):

This manuscript presents a clear and rigorous reanalysis of three patch-leaving datasets (two human, one rat) to test whether stochastic decision rules alone can account for observed variability in patch-leaving times. The authors show that a simple stochastic choice model with reward-dependent (varying over conditions) and reward-independent components (fixed over reward conditions) can reproduce patch-leaving behavioural variability across species and tass. A secondary contribution is the analysis of internal models (M1–M4) relating average reward rate to reward weighting. Across the three datasets, the authors find generalisability of model predictions. The reproductions of behavioural variability by the models overlaid in comparison to that of participants make for some striking plots. There wasn't any pre-registration that I saw, though data and source code appear open and available publicly on GitHub.

Overall, this is solid, careful, and technically competent work. The paper convincingly addresses its central question and the analyses seem sound. The writing is clear and the figures are excellent. I recommend publication subject only to minor revisions to improve clarity and interpretation.

Major Points (all minor in severity)
1. Clarify the rationale for the two-stage model-fitting strategy

The paper first uses "vary c / vary B / vary both" models to account for condition effects and then carries forward the winning variant (fix c vary B) to examine individual differences and internal models (M1–M4). Though I sympathise with the authors' probable desire for a tractable model space, is reasonable for tractability, is it not a more rigorous procedure to all parameters to vary for both condition-level and participant-level effects in a single unified model space? Perhaps it will help the reader if the authors briefly justify their choice of modelling pipeline.

2. Model comparison for M1–M4 feels under-justified

Section on models M1–M4 reports somewhat inconsistent patterns across datasets (e.g., M1 preferred in field-human, M4 in the other two and then a different result when counting number of best fitting participants per model in the supplementary materials). This is not necessarily a problem. The authors appear to appropriately refrain from over-interpreting it, though neither does the write-up acknowledge the inconsistency and explain how strongly (or weakly) these results should influence interpretation.

In my view, the main message of the paper doesn't hinge on identifying a single best internal model in this specific model comparison, but a brief paragraph explicitly stating the role these comparisons play and the limits of the authors' conclusions might help avoid over-interpretation by readers.

3. What really is important about the different M1-M4 models? The four different ways of computing B are shown with minimal background or discussion. This is understandable in a sense because I can see that they're mathematically

sensible and simple ways of transforming the reward rate. But if one model is preferred over another in model comparison, then just what is the theoretical importance of that? What are the theoretical implications of each?

Reviewer #3 (Remarks to the Author):

A widely used optimal model of patch-foraging is the Marginal Value Theorem, which assumes a deterministic decision rule. Using three previously published datasets, the authors claim that foragers, both human and rat, exhibit substantial intra-individual variability in their patch-leaving times. I believe the authors provide substantial evidence supporting this claim. Strengths of the paper include the use of multiple datasets spanning different species, converging results, strong model recovery and parameter recovery, and solid model validation. My main concern, however, is that it is already very common in the patch-foraging and broader decision-making literature to model agent choice as stochastic (e.g., using a softmax decision rule rather than a purely greedy strategy). As a result, the novelty of the contribution seems limited. That said, I may have misunderstood the authors' goals and welcome clarification on this point. Below I outline additional concerns.

Major concerns:
1. It would be helpful if the authors provided more motivation for their research question in the introduction. Why should we care about intra-individual variability in patch-leaving times beyond how it differs from Marginal Value Theorem? What is the literature on intra-individual variability in the decision-making literature more generally?

2.The authors find an effect of environment richness on variability only in the experiment with linearly decaying rewards. They claim that the linearly-decaying rewards "cause" variability to be sensitive to environmental richness. However, could this also be driven by the average stochasticity parameter, B, which for rates is about 0.10? If I'm interpreting their plots correctly, based on their model predictions (Fig. 4a-c), foragers with B around 0.1 should show sensitivity to environmental richness under both exponential and linear decay.

3. There is a substantial difference in BIC between the vary-B-fix-C model and the fix-B-fix-C model. However, in Figure S3, they appear to capture behavior similarly well. What accounts for the difference in BIC?

4. The authors provide model and parameter recovery results for models that vary or fix B and C, but not for the models M1-M4. Which differ in how the average reward rate of the environment is used to adjust B. Please include recovery analyses for these models as well.

5. Figure S3 demonstrates that the model captures individual differences across participants, but it is difficult to assess the quantitative fit here. Please provide plots showing the distribution of differences between predicted and observed leaving times. This would make it easier to assess whether the model systematically under-or over-estimates leaving times.

Minor concerns:
1. paper uses the coefficient of variation (CV) in the first results section, but switches to standard deviation later. Why not continue with CV throughout, especially because it takes into account differences in the mean? The standard deviation of leaving times is similar across environments and patch types, but the CV does appear to differ, at least across patch types in the field-human data.

2. I am not sure the phrase "internal model of the environment" is justified here. The model assumes that the foragers track the overall richness of the environment and adjust B accordingly. This doesn't require having a rich internal model that contains the relationships between states and events. The authors could either provide their definition of an "internal model" or substitute it for another phrase.

* TRANSPARENT PEER REVIEW: Communications Psychology uses a transparent peer review system. This means that we publish the editorial decision letters including Reviewers' comments to the authors and the author rebuttal letters online as a supplementary peer review file. However, on author request, confidential information and data can be removed from the published reviewer reports and rebuttal letters prior to publication. If your manuscript has been previously reviewed at another journal, those Reviewers' comments would not form part of the published peer review file.

** Visit Nature Research's author and referees' website at www.nature.com/authors for information about policies, services and author benefits**

Communications Psychology is committed to improving transparency in authorship. As part of our efforts in this direction, we are now requesting that all authors identified as 'corresponding author' create and link their Open Researcher and Contributor Identifier (ORCID) with their account on the Manuscript Tracking System prior to acceptance. ORCID helps the scientific community achieve unambiguous attribution of all scholarly contributions. You can create and link your ORCID from the home page of the Manuscript Tracking System by clicking on 'Modify my Springer Nature account' and following the instructions in the link below. Please also inform all co-authors that they can add their ORCIDs to their accounts and that they must do so prior to acceptance.

https://www.springernature.com/gp/researchers/orcid/orcid-for-nature-research

For more information please visit http://www.springernature.com/orcid

If you experience problems in linking your ORCID, please contact the Platform Support Helpdesk.

Version 1:

Decision Letter:

** Please ensure you delete the link to your author homepage in this e-mail if you wish to forward it to your coauthors **

Dear Dr Scholey,

Your manuscript titled "Stochastic choice drives variability in patch foraging decisions in humans and rats" has now been seen by our reviewers, whose comments appear below. In light of their advice I am delighted to say that we are happy, in principle, to publish a suitably revised version in Communications Psychology.

We therefore invite you to revise your paper one last time to address the remaining concerns of our reviewers and a list of editorial requests. At the same time we ask that you edit your manuscript to comply with our format requirements and to maximise the accessibility and therefore the impact of your work.

EDITORIAL REQUESTS:

Please review our specific editorial comments and requests regarding your manuscript in the attached "Editorial Requests Table". Please outline your response to each request in the right hand column. Please upload the completed table with your manuscript files as a Related Manuscript file.

If you have any questions or concerns about any of our requests, please do not hesitate to contact me.

SUBMISSION INFORMATION:

In order to accept your paper, we require the files listed here https://www.nature.com/documents/commsj-file-checklist.pdf .

OPEN ACCESS:

Communications Psychology is a fully open access journal. Articles are made freely accessible on publication. For further information about article processing charges, open access funding, and advice and support from Nature Research, please visit https://www.nature.com/commspsychol/open-access

At acceptance, you will be provided with instructions for completing the open access licence agreement on behalf of all authors. This grants us the necessary permissions to publish your paper. Additionally, you will be asked to declare that all required third party permissions have been obtained, and to provide billing information in order to pay the article-processing charge (APC).

* TRANSPARENT PEER REVIEW: Communications Psychology uses a transparent peer review system. On author request, confidential information and data can be removed from the published reviewer reports and rebuttal letters prior to publication. If you are concerned about the release of confidential data, please let us know specifically what information you would like to have removed. Please note that we cannot incorporate redactions for any other reasons.

* CODE AVAILABILITY: All Communications Psychology manuscripts must include a section titled "Code Availability" at the end of the methods section. We require that the custom analysis code supporting your conclusions is made available in a publicly accessible repository at this stage; please choose a repository that generates a digital object identifier (DOI) for the code; the link to the repository and the DOI must be included in the Code Availability statement. Publication as Supplementary Information will not suffice.

* DATA AVAILABILITY:
All Communications Psychology manuscripts must include a section titled "Data Availability" at the end of the Methods section. More information on this policy, is available in the Editorial Requests Table and at http://www.nature.com/authors/policies/data/data-availability-statements-data-citations.pdf.

Please use the following link to submit the above items:
Link Redacted
** This url links to your confidential home page and associated information about manuscripts you may have submitted or be

reviewing for us. If you wish to forward this email to co-authors, please delete the link to your homepage first **

We hope to hear from you within two weeks; please let us know if you need more time.

Best regards,

Troby Lui, on behalf of
Eva R. Pool

Troby Lui, PhD
Associate Editor
Communications Psychology

Eva R. Pool, PhD
Editorial Board Member
Communications Psychology
orcid.org/0000-0001-5929-1007

REVIEWERS' COMMENTS:

Reviewer #1 (Remarks to the Author):

Thank you for addressing my concerns. I have no further comments.

Reviewer #2 (Remarks to the Author):

Thanks to the authors for responding to my comments.

Reviewer #3 (Remarks to the Author):

Thank you to the authors for their thoughtful responses. They have satisfactorily addressed my concerns. I have no further comments.

** Visit Nature Research's author and referees' website at www.nature.com/authors for information about policies, services and author benefits**

Open Access This Peer Review File is licensed under a Creative Commons Attribution 4.0 International License, which permits use, sharing, adaptation, distribution and reproduction in any medium or format, as long as you give appropriate credit to the original author(s) and the source, provide a link to the Creative Commons license, and indicate if changes were made.
In cases where reviewers are anonymous, credit should be given to 'Anonymous Referee' and the source.
The images or other third party material in this Peer Review File are included in the article's Creative Commons license, unless indicated otherwise in a credit line to the material. If material is not included in the article's Creative Commons license and your intended use is not permitted by statutory regulation or exceeds the permitted use, you will need to obtain permission directly from the copyright holder.
To view a copy of this license, visit https://creativecommons.org/licenses/by/4.0/

Reviewer comments for Comms Psych submission: Foraging variability manuscript

We thank the reviewers for their comments. We detail our response to each point below. In addition to the revisions in response to these comments, we have made the following changes to align with the editorial requests in the mandatory Nature Communications Psychology manuscript checklist, including:

- **Title**: changed to "Stochastic choice drives variability in patch foraging decisions in humans and rats" to identify the species we study
- **Abstract**: included the number of participants and the species studied for each dataset
- **General**: rearranged the order so that Methods precedes Results; added a Limitations subheading in the Discussion; rephrased all MVT and RL acronyms to 'marginal value theorem' and 'reinforcement learning' respectively
- **Introduction**: edited the final paragraph of the introduction to only describe our model predictions/hypotheses, and removed description of empirical results.
- **Methods**: added preregistration and ethics information; added demographics information where possible for each dataset; added details on how confidence intervals were calculated, and that we checked assumptions for statistical tests
- **Results:** added sample numbers in every figure caption; added confidence intervals and effect sizes for all statistical tests
- **End matter**: separated data and code availability statements; included competing interests and acknowledgements statements.
- **Graphs**: edited Figures 4d-f, 5a-b, 6a,c to show y-axis from 0. For Figures 5a and 6a, we now plot the y-axis as the difference between each models' BIC and the BIC of the highlighted winning model, so that the axes intersect 0. We have edited the legends appropriately.
- **Supplementary**: added confidence intervals in Tables S1 and S2.

The line numbers below refer to the version of the manuscript with tracked changes. We have also provided a clean version of the manuscript to aid readability.

<u>Reviewer #1</u>:

R1.0 This paper tests a stochastic choice model to model variability in patch-leaving decisions in various foraging tasks, across humans and rats. I really appreciate the idea of comparing across similar experiments and species to understand the fundamentals of decision-making. However, I found the motivations for the paper and the models, presentation of results and the implications to be lacking. Mainly, I am not sure I follow the authors' main claim that environments don't predict choice variability. It is confusing to tell whether certain environments have a greater variability or not, due to the use of two variables (CV and SD), and the model explanations are also hard to follow. I would suggest the authors to edit substantially

to improve the readability of the manuscript, while clarifying why these questions matter, why certain models were used and what the results mean.

Response: Thank you very much for your comments and suggestions on readability and presentation of results. Your feedback has helped us to clarify our presentation of CV and SD to highlight variability in choice. We have also elaborated on the gap in the literature and implications of our results.

R1.1 Abstract could frame the gap or the question better. What is stochastic choice? Why do we care?

Response: We have added text to the abstract on lines 5-9 and lines 17-18 to introduce the reader to the concept of stochastic choice, and why it is interesting to study in a foraging context.

R1.2 Figure 1 is quite nice! One suggestion would be to mention what the %mean in (b) in the legend.

Response: Thank you. We have added this to the legend of Figure 1.

R1.3 If the demarcation between early and later trials were to be changed, will the results in Fig 2(b) remain the same? If foragers can learn quickly, then early trials need not be the first half, but rather first 3rd or 4th of all trials. If the authors plot the CV_leave over time, and see whether there is a change point after initial trials, then that would present a clearer picture. This is especially important because theoretically variation in such decisions indicate learning.

Response: Our decision to split the block in half for our comparisons was to ensure we had a sufficient number of trials within each block split to make robust statistical inferences of the standard deviation. A more fine-grained demarcation (e.g. thirds or fourths within a block) results in insufficient trial counts to make a robust inference (i.e. some subjects have as little as 2 trial counts for calculating standard deviation for the same patch type).

Despite these concerns, we divided the block into thirds so we might examine participants' variation in the first third of trials, as the reviewer suggested. Please note these plots use SD-leave as a measure of variability, rather than CV-leave, in response to comments R1.5-8 and R1.10 below. It is reassuring to see that SD-leave remains relatively consistent across each third for all of the datasets. Overall, these plots support our claim that variability remains unchanged across the blocks:

Reviewer comments for Comms Psych submission: Foraging variability manuscript

Field-human:

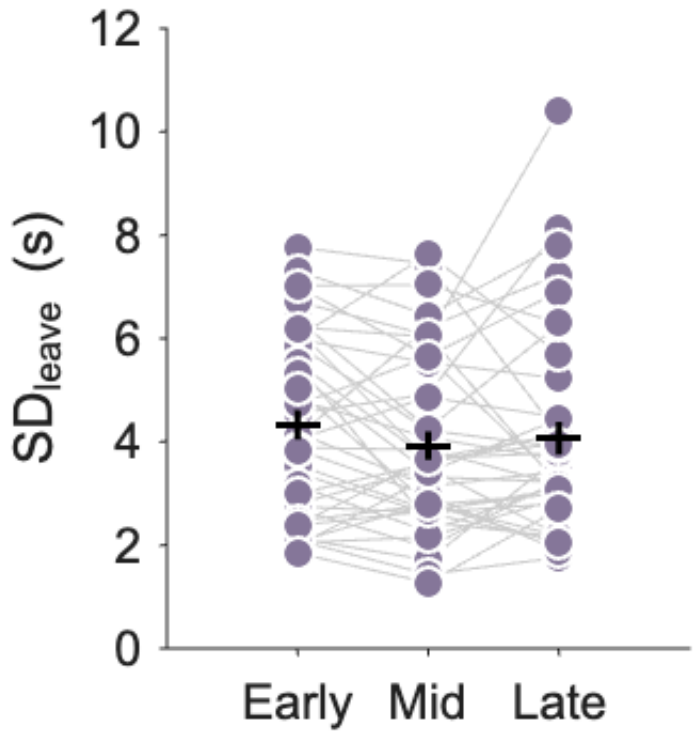

Berry-human:

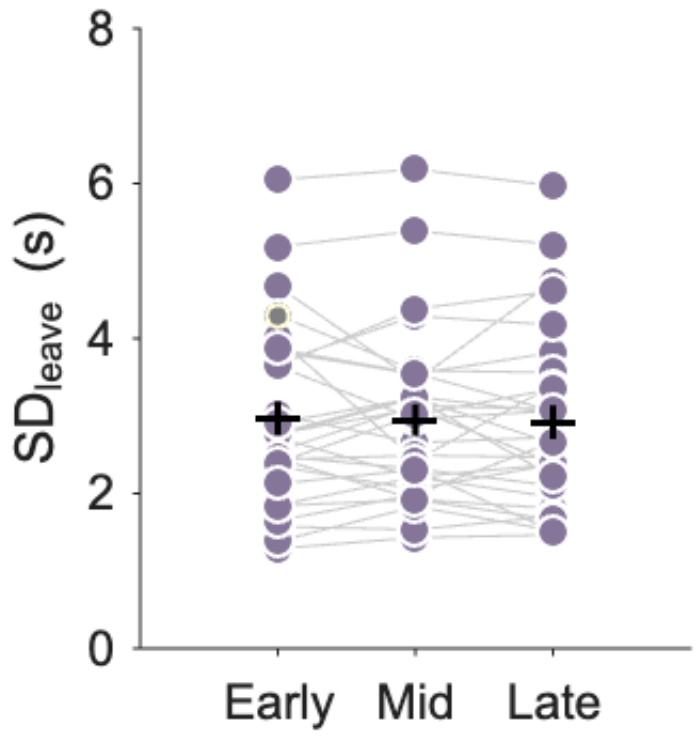

Rat:

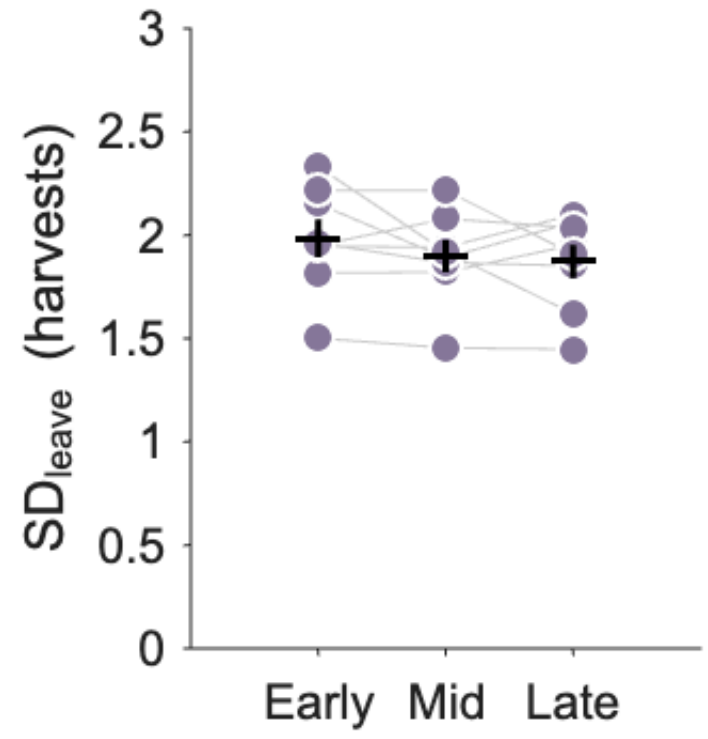

We have not conducted statistical inference on these results, and we do not plan to include these plots in the manuscript, due to the trial count concerns expressed above.

R1.4 Relatedly, what is the direction of the CV_leave in terms of overstaying or understaying? could it be that the direction is the meaningful axis that demarcates CV differences between early and late trials -- people could vary from the mean to overstay in early trials, but in later trials they vary more in understaying.

Response: This is an interesting hypothesis. Overstaying or understaying are relative to the optimal times predicted by the marginal value theorem (MVT). Since MVT is normative, it predicts that foragers will not show any variation: optimal foragers should consistently leave the same patch in the same environment at the identical optimal leaving time. Therefore, there is no concept of over or understaying for variation of leaving times (SD-leave).

However, we can look at the mean leaving times compared to MVT, and how these change as a function of early vs late phases, to examine whether foragers underharvest in later phases. These plots are shown below, with the average optimal MVT leaving time across patches/environments shown by the dotted line:

Field-human:

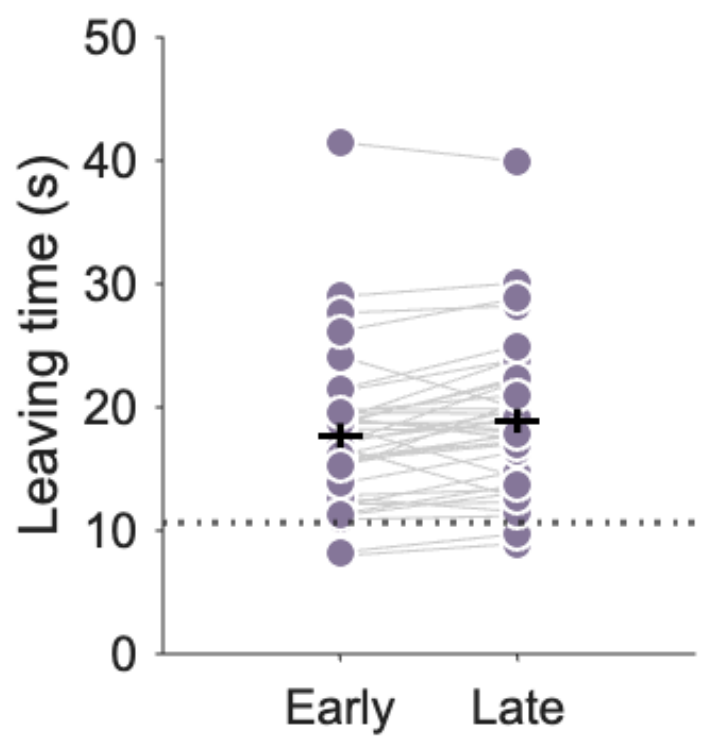

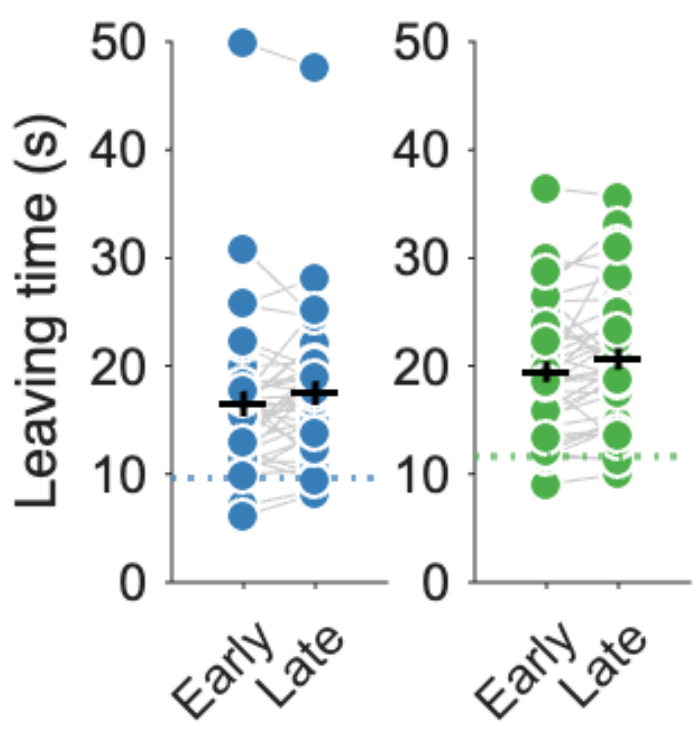

Reviewer comments for Comms Psych submission: Foraging variability manuscript

Berry-human:

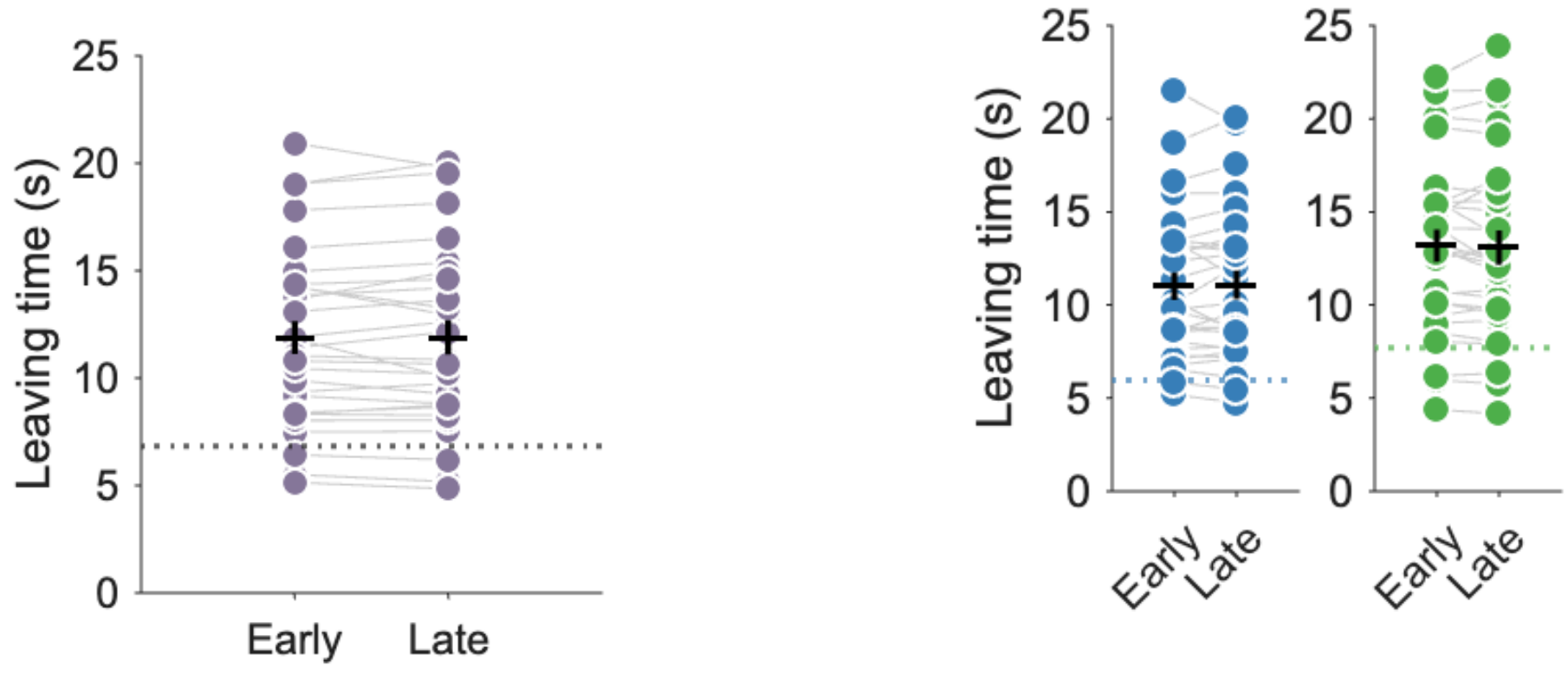

Rat:

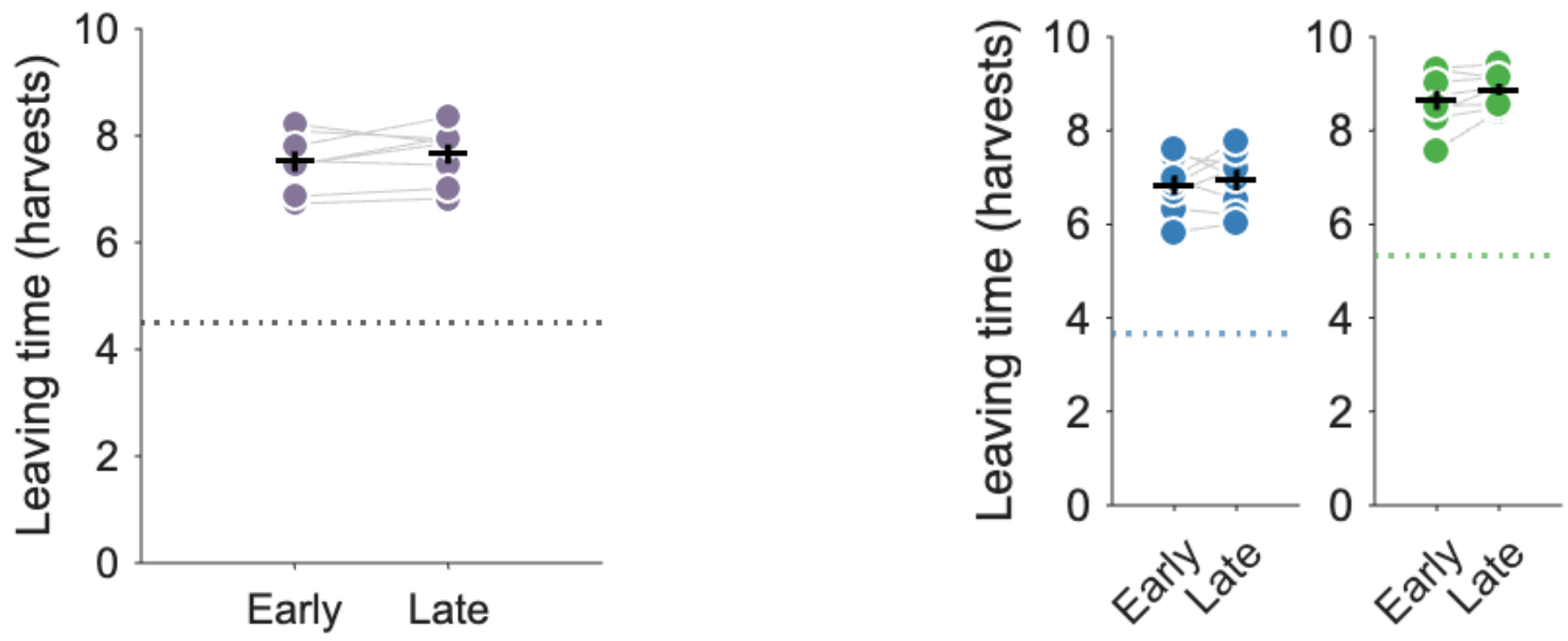

These plots indicate that mean leaving times are (1) broadly similar across early and late phases and (2) consistently show overharvesting across early and late phases. Therefore these results suggest that overharvesting does not meaningfully differ across early and late phases.

Since overharvesting is not the subject of this paper, we do not believe it is beneficial to add these plots to the manuscript.

R1.5 Discussion of Fig2a is lacking on Page 6. CV seems to be highest for the incongruent condition of rich environments + low yield, across the 3 tasks, although it is more pronounced in the rat task. This pattern has not been commented upon. This is crucial because this effect is what the authors are trying to suggest their model speaks against (i.e., environment does not explain variability).

Response: In the original manuscript, we chose not to discuss CV-leave in Figure 2a in detail as CV-leave was not our main measure of model-predicted variability; we used SD-leave as our variability metric. We did not comment on the pattern of

CV-leave because this pattern could either be driven by the numerator (SD-leave) or the denominator (mean leaving times). Mean leaving times showed a strong environment/patch dependence, as shown in Figure 1d. Therefore, the patterns of change in CV-leave were entirely or mostly driven by changes in the mean, not the variability, of leaving times.

Our intention for using CV-leave instead of SD-leave in Figure 2a was to show the reader that participants' variability was high: it was a large proportion of their mean leaving time. However, we realise that this causes confusion when trying to reconcile Figure 2a with the later SD-leave results in Figure 4 (i.e. that environment does not necessarily explain variability). We have decided to remove Figure 2a and replace it with panels showing the distribution of SD-leave as a function of patch and environment. These panels show that SD-leave is relatively high across all combinations of patch types and environments.

We have also replaced Figure 2b with plots showing SD-leave in early and late trials, rather than CV-leave.

We have updated the statistical results starting from line 359 comparing SD_leave between early and late trials in an environment; the conclusions of these results remain the same as before, that SD-leave does not change between early and late trials.

In our original manuscript, we found a small but significant difference in CV-leave for early vs late trials for the field-human dataset only. To identify whether this difference was specific to rich or poor environments, we had originally plotted in Figure S1b the CV-leave in early vs late trials, separated by environment. Given that we now find no significant difference in SD-leave for the field-human dataset (or across any of the datasets), we have removed Figure S1b.

R1.6 In addition, the 3 tasks do have differences. the rat task has a 3-fold difference between the travel times, while the berry task has a very minimal difference.

Response: Thank you for highlighting the travel time difference between the berry and rat task. We have added text on line 338 to make the difference in travel time magnitude clear to the reader.

R1.7 The results from the models are hard to follow. Why did the authors show the CV earlier and then switch to SD in Fig 3 and 4? Around L181, the authors say that variability is independent of the type of patch/environment. This is quite odd because the behavioral results show clearly that rich environment-low yield patches have the highest CV.

Response: We have dealt with this point as detailed in the response to R1.5 above, by removing CV-leave plots. Our claim that variability is independent of patch type or environment is evidenced by Figure 4, where subjects' empirical SD-leave is broadly consistent across patches and environments, as predicted by the model. We hope that by removing the CV-leave plots, whose differences were driven by the mean leaving time, this is easier to follow.

R1.8 Plus, in the line 183, the authors say that reward weighting between environments provides a better account. This would mean that sensitivity to the environment matters, right? It is difficult for me to follow the model and the results because of the way they are written, but the inferences are doubtful.

Response: The reward weighting parameter varying by environments does provide a better account, as evidenced by model fitting (Figure 5a) and the fact that the reward weighting parameter was significantly higher for poor compared to rich environments (Figure S6d). This means that sensitivity to the environment is important. However, this weighting parameter captures the environment sensitivity for mean leaving times i.e. that subjects leave patches in rich environments earlier than in poor environments (Figure 1d). Even though the reward weighting parameter is environment-dependent, this does not necessarily predict that variability is environment dependent, as shown in Figure 4a-c. The fitted values of the reward weighting parameter for the human task places their SD-leave in the plateau predicted by our model for both the poor and rich environments; consequently SD-leave does not change as a function of environment. We explain this on lines 437 - 444 and lines 449-454.

We realise that these predictions are quite counter-intuitive, because they are quite different to what is predicted in simultaneous choice tasks. We outline this in the discussion from line 562-570.

We appreciate that some of this confusion may have come from presenting the CV-leave plots first in Figure 2, and thus hope that removing the CV-leave plots makes our SD-leave inferences clearer.

R1.9 Fig 3. Why is the bias, c set to -3.6? and why does it show only negative sweeps of c? If people and rats tend to usually overstay in a patch, then the bias should be > 0, right?

Response: We selected a value of c=-3.6 to illustrate a slice through the heat-maps of Figure 3, to aid their interpretation. The heatmaps in Figure 3 show that setting any value of c would give qualitatively the same dependence of SD-leave on the reward-weighting B as is shown in that slice (pink line) for each task.

For the second point, the heatmaps show mostly negative sweeps of c because positive c produces leaving times that are markedly longer than most subjects' leaving times. Looking at Figure 5c, only a handful of participants across the human tasks show a positive c, and the ranges observed in subject data are the ranges we generate the heatmap predictions for in Figure 3.

The reviewer's inference that bias should be >0 when overstaying likely stemmed from line 385, "*[c]...can be either negative (favouring leaving), positive (favouring staying) or zero…*". The action of c is complex due to its interactions with B; its function is to change the bias in leaving times with regard to where B is set. This means that the action of c is not necessarily symmetrical around 0. To avoid confusing the reader, we have replaced line 385 outlined above with "*... more negative values [of c] favouring leaving…*".

R1.10 L194-98. This is confusing - Fig 2 showed that rich environments+low yield for the rat task had the highest variability, now based on the SD, the authors say that low-yield patches have the least variability.

Response: We have dealt with this point as detailed in the response to R1.5 above, by removing CV-leave plots.

R1.11 There are many grammatical errors in the manuscript, some below:
72. "were inspired"
73. "types of patches", perhaps
81-82. "the patch effect" and "the environment effect" are written in a way suggesting that these effects by these names have been previously defined, which they have not.
112. I know the hypothesis is stated in the previous line but because of the subsection change and the figure, it doesn't stick. Would help to reiterate.
145. "depend on". the sentence reads odd.
171. "all model variants" or "all models"

Response: Thank you, these have been corrected.

Reviewer #2 (Remarks to the Author):

R2.0 This manuscript presents a clear and rigorous reanalysis of three patch-leaving datasets (two human, one rat) to test whether stochastic decision rules alone can account for observed variability in patch-leaving times. The authors show that a simple stochastic choice model with reward-dependent (varying over conditions) and reward-independent components (fixed over reward conditions) can reproduce patch-leaving behavioural variability across species and tass. A secondary contribution is the analysis of internal models (M1–M4) relating average reward rate to reward weighting. Across the three datasets, the authors find generalisability of

model predictions. The reproductions of behavioural variability by the models overlaid in comparison to that of participants make for some striking plots. There wasn't any pre-registration that I saw, though data and source code appear open and available publicly on GitHub.

Overall, this is solid, careful, and technically competent work. The paper convincingly addresses its central question and the analyses seem sound. The writing is clear and the figures are excellent. I recommend publication subject only to minor revisions to improve clarity and interpretation.

Response: Thank you for your supportive comments and constructive feedback on our manuscript.

Major Points (all minor in severity)
R2.1 Clarify the rationale for the two-stage model-fitting strategy

The paper first uses "vary c / vary B / vary both" models to account for condition effects and then carries forward the winning variant (fix c vary B) to examine individual differences and internal models (M1–M4). Though I sympathise with the authors' probable desire for a tractable model space, is reasonable for tractability, is it not a more rigorous procedure to all parameters to vary for both condition-level and participant-level effects in a single unified model space? Perhaps it will help the reader if the authors briefly justify their choice of modelling pipeline.

Response: As you mentioned, we first compared "vary c / vary B / vary both" models, and then carried forward the winning variant (fix c, vary B) into the internal model analyses (M1–M4). If we understand correctly, the question is whether it would be more robust to assume at the outset that c varies as a function of average reward, and therefore allow both c and B to vary as a function of an internal model of average reward rate. We could then analyse all models together in a single model space rather than in a two-step process. Our reasons for not doing this and using a two-step modelling pipeline are as follows.

First, our goal in the first modelling step was to derive clear, testable predictions for how specific mechanisms (reward-independent bias c vs reward-dependent weighting B) shape the variation in leaving times (SDleave) across patches and environments. Allowing both c and B to vary with internal models from the outset would create a large, flexible model space in which multiple combinations of parameters could reproduce the same pattern of mean leaving times. However, this would make it much harder to see which mechanism actually generates the observed variability patterns (e.g. the environment-independent SDleave in the human tasks and the inverted environment effect in rats). By first identifying whether variability is better captured by changing B or c between environments, we can

determine if reward-independent or reward-dependent mechanisms influence stochastic choice.

Second, our first model comparison results (Fig 5) suggest that internal models of c would give poor fits to the data, as the "vary B, fix c" model systematically outperformed alternatives across tasks. This suggests that c is approximately constant across environments and is not a valid target for an internal model of average reward, whereas B is.

Finally, a unified model space in which both c and B are allowed to vary with internal models of average reward would be weakly identifiable from the kind of data available here, with more parameters leading to higher risk of parameter trade-off. Therefore different internal models (for c vs B) would be indistinguishable but lead to different mechanistic interpretations.

For these reasons, we believe our current modelling pipeline keeps the model space tractable, allows interpretation of SDleave in terms of clear mechanisms, and avoids drawing strong conclusions from a large model space.

We have now added text in the Methods section (lines 227 - 232 and lines 270-274), being more explicit about this two-step process and our reasons for it.

R2.2 Model comparison for M1–M4 feels under-justified

Section on models M1–M4 reports somewhat inconsistent patterns across datasets (e.g., M1 preferred in field-human, M4 in the other two and then a different result when counting number of best fitting participants per model in the supplementary materials). This is not necessarily a problem. The authors appear to appropriately refrain from over-interpreting it, though neither does the write-up acknowledge the inconsistency and explain how strongly (or weakly) these results should influence interpretation.

In my view, the main message of the paper doesn't hinge on identifying a single best internal model in this specific model comparison, but a brief paragraph explicitly stating the role these comparisons play and the limits of the authors' conclusions might help avoid over-interpretation by readers.

What really is important about the different M1-M4 models? The four different ways of computing B are shown with minimal background or discussion. This is understandable in a sense because I can see that they're mathematically sensible and simple ways of transforming the reward rate. But if one model is preferred over another in model comparison, then just what is the theoretical importance of that? What are the theoretical implications of each?

Response: Thank you for this thoughtful point. We agree that the central claim of the paper does not depend on a single "winning" internal model. Our goal for this part of the paper was to show that there can exist a systematic mapping from average reward rate to the reward-weighting parameter. Thus our intention was to not interpret the "best" model, but to show there was a good internal model that could account for the data; we used model selection to choose which model as our exemplar of a good one. We have added text in the Results section on lines 523-526 to make our goal of this model selection clear to the reader, and added lines 532-535 to discuss the inconsistencies in the winning model across datasets and how we interpret this.

We have also acknowledged that there are no strong theoretical implications of the four exemplar models when we introduce them in the Methods (line 278-280) and discuss the limitations of this approach in the Discussion (line 623 - 629).

Reviewer #3 (Remarks to the Author):

R3.0 A widely used optimal model of patch-foraging is the Marginal Value Theorem, which assumes a deterministic decision rule. Using three previously published datasets, the authors claim that foragers, both human and rat, exhibit substantial intra-individual variability in their patch-leaving times. I believe the authors provide substantial evidence supporting this claim. Strengths of the paper include the use of multiple datasets spanning different species, converging results, strong model recovery and parameter recovery, and solid model validation. My main concern, however, is that it is already very common in the patch-foraging and broader decision-making literature to model agent choice as stochastic (e.g., using a softmax decision rule rather than a purely greedy strategy). As a result, the novelty of the contribution seems limited. That said, I may have misunderstood the authors' goals and welcome clarification on this point. Below I outline additional concerns.

Response: Thank you for your feedback that the central claims of the paper are supported by multiple datasets and strong modelling approaches, we appreciate this. We have taken on board your comments about the novelty, and provided more context in the introduction about why it is beneficial to look at variability in foraging.

Major concerns:

R3.1 It would be helpful if the authors provided more motivation for their research question in the introduction. Why should we care about intra-individual variability in patch-leaving times beyond how it differs from Marginal Value Theorem? What is the literature on intra-individual variability in the decision-making literature more generally?

Reviewer comments for Comms Psych submission: Foraging variability manuscript

Response: Thank you for this feedback. We have added substantial text to the introduction (lines 51 - 65) to expand on the novelty, our motivation and the wider literature.

R3.2 The authors find an effect of environment richness on variability only in the experiment with linearly decaying rewards. They claim that the linearly-decaying rewards “cause” variability to be sensitive to environmental richness. However, could this also be driven by the average stochasticity parameter, B, which for rates is about 0.10? If I’m interpreting their plots correctly, based on their model predictions (Fig. 4a-c), foragers with B around 0.1 should show sensitivity to environmental richness under both exponential and linear decay.

Response: The reviewer is absolutely correct that if foragers in the human tasks had a B of around 0.1, then our model predicts their variability will be sensitive to environmental richness. However, fitting the model to participants’ leaving times in both tasks showed B is higher [Fig S6d]. This predicted their variability of leaving would have no environmental dependence, which is what we observe in the empirical data [Fig 4].

We have added further text about the model’s predictions of environmental sensitivity at lower B to the discussion (Lines 630-639).

R3.3 There is a substantial difference in BIC between the vary-B-fix-C model and the fix-B-fix-C model. However, in Figure S3, they appear to capture behavior similarly well. What accounts for the difference in BIC?

Response: It is correct that there is a large difference in BIC between the ‘vary B fix C’and ‘fix B fix C’ model. However, we have not shown in Fig S3 that they capture behaviour similarly well because we do not include the ‘fix B fix C’ control model in that figure. This model cannot capture the majority of participants’ behaviour as it predicts no environment effect on leaving times (Figure S2). We do find in our model comparison that a minority of individuals are better fit by the fix c fix B model, as we explain on lines 488-495.

R3.4 The authors provide model and parameter recovery results for models that vary or fix B and C, but not for the models M1-M4. Which differ in how the average reward rate of the environment is used to adjust B. Please include recovery analyses for these models as well.

Response: We agree that model and parameter recovery are an important component of model selection when determining the best fitting model. However, we believe that this step only matters when wanting to make strong claims that one

model outperforms the other, such as in Figure 5 when comparing the fix/vary B/c models. As highlighted by reviewer 2 in point R2.2, we do not make a strong claim that any of M1-4 is the 'correct' candidate model. Rather, we tested that a potential mapping linking B to average reward can provide a good account of behaviour on our tasks. In theory, this mapping could take many forms. Thus we believe that model and parameter recovery would be misleading, as it would imply we seek to identify and propose a single form of the mapping; we also note that changes to these model comparisons would not change any of the core conclusions of our paper: they depend only on the model fits being a good account of behaviour, not which one is the best.

In response to R2.2 and this feedback point, we have now added text to inform the reader of our goals for the M1-M4 model selection step on lines 523-526. We also discuss the limitations of this model selection step explicitly on lines 623-629 in the discussion.

R3.5 Figure S3 demonstrates that the model captures individual differences across participants, but it is difficult to assess the quantitative fit here. Please provide plots showing the distribution of differences between predicted and observed leaving times. This would make it easier to assess whether the model systematically under-or over-estimates leaving times.

Response: Thank you for this suggestion. We have generated these plots and added them to Figure S3. They suggest excellent quantitative fit, and no systematic under or over-estimation of leaving times across environments. We have included text on lines 424 - 426 to highlight this to the reader.

Minor concerns:

R3.6 paper uses the coefficient of variation (CV) in the first results section, but switches to standard deviation later. Why not continue with CV throughout, especially because it takes into account differences in the mean? The standard deviation of leaving times is similar across environments and patch types, but the CV does appear to differ, at least across patch types in the field-human data.

Response: We chose not to continue with CV throughout, precisely because it takes into account differences in the mean. The interesting patterns in variability (SD-leave) that we observe would be masked by the mean leaving times, which show a strong modulation by environment and patch (Figure 1d).

We appreciate switching from CV-leave to SD-leave was potentially confusing, and this was also highlighted by Reviewer 1. We have therefore decided to change the results in Figure 2 to show SD-leave, so that this is consistent throughout. Specifically, we have changed Figure 2a to show the distributions of participants' of SD-leave across different environments and patch types, and Figure 2b to show

SD-leave in early versus late trials. We have updated the statistical results on lines 356 - 371 comparing SD-leave in early vs late trials; the conclusions of these results remain the same as before, that SD-leave does not change between early and late trials.

R3.7 I am not sure the phrase "internal model of the environment" is justified here. The model assumes that the foragers track the overall richness of the environment and adjust B accordingly. This doesn't require having a rich internal model that contains the relationships between states and events. The authors could either provide their definition of an "internal model" or substitute it for another phrase.

Response: To avoid potential confusion over what does and does not count as an internal model, we have changed 'internal model' to 'internal function' throughout.
