# Supplementary material for: Stochastic choice drives variability in patch foraging decisions in humans and rats
Source: Commun Psychol. 2026 May 19;4:112. doi: 10.1038/s44271-026-00465-0 (PMC13447090; doi:10.1038/s44271-026-00465-0)
Supplement: Supplementary file 3 [file 44271_2026_465_MOESM3_ESM.pdf]

nature portfolio

Corresponding author(s): Dr Emma Scholey and Professor Mark Humphries

Last updated by author(s): Apr 3, 2026

Reporting Summary

Nature Portfolio wishes to improve the reproducibility of the work that we publish. This form provides structure for consistency and transparency in reporting. For further information on Nature Portfolio policies, see our Editorial Policies and the Editorial Policy Checklist.

Statistics

For all statistical analyses, confirm that the following items are present in the figure legend, table legend, main text, or Methods section.

n/a	Confirmed	
☐	☒	The exact sample size (*n*) for each experimental group/condition, given as a discrete number and unit of measurement
☐	☒	A statement on whether measurements were taken from distinct samples or whether the same sample was measured repeatedly
☐	☒	The statistical test(s) used AND whether they are one- or two-sided<br>*Only common tests should be described solely by name; describe more complex techniques in the Methods section.*
☐	☒	A description of all covariates tested
☐	☒	A description of any assumptions or corrections, such as tests of normality and adjustment for multiple comparisons
☐	☒	A full description of the statistical parameters including central tendency (e.g. means) or other basic estimates (e.g. regression coefficient) AND variation (e.g. standard deviation) or associated estimates of uncertainty (e.g. confidence intervals)
☐	☒	For null hypothesis testing, the test statistic (e.g. *F*, *t*, *r*) with confidence intervals, effect sizes, degrees of freedom and *P* value noted<br>*Give P values as exact values whenever suitable.*
☐	☒	For Bayesian analysis, information on the choice of priors and Markov chain Monte Carlo settings
☐	☒	For hierarchical and complex designs, identification of the appropriate level for tests and full reporting of outcomes
☐	☒	Estimates of effect sizes (e.g. Cohen's *d*, Pearson's *r*), indicating how they were calculated

Our web collection on statistics for biologists contains articles on many of the points above.

Software and code

Policy information about availability of computer code

Data collection	Existing data were used from Le Heron et al (2020) https://doi.org/10.1523/JNEUROSCI.2586-19.2020 for the field-human task; Contreras-Huerta et al (2024) https://doi.org/10.1038/s41598-024-69452-x for the berry-human task; Kane et al (2017) https://doi.org/10.7554/eLife.48429 for the rat task. The original data for the field-human and berry-human tasks were collected using the Psychophysics Toolbox (version not reported in original article) for MATLAB (version not reported in original article). There was no software reported in the original article for the rat task.
Data analysis	Behavioural data were analysed in R (v4.4.1) using the lme4 package (v1.1-35.4) and BayesFactor package (v0.9.12-4.7) , and JASP (v0.19.3). Computational modelling was conducted in MATLAB (v2023b) using custom code. Analysis scripts and raw data can be found on GitHub (https://github.com/Humphries-Lab/Foraging-variability).

For manuscripts utilizing custom algorithms or software that are central to the research but not yet described in published literature, software must be made available to editors and reviewers. We strongly encourage code deposition in a community repository (e.g. GitHub). See the Nature Portfolio guidelines for submitting code & software for further information.

Data

Policy information about availability of data

All manuscripts must include a data availability statement. This statement should provide the following information, where applicable:

- Accession codes, unique identifiers, or web links for publicly available datasets
- A description of any restrictions on data availability
- For clinical datasets or third party data, please ensure that the statement adheres to our policy

All code and source data used to produce the figures in this paper are available from GitHub (https://github.com/Humphries-Lab/Foraging-variability). Original source data for the berry-human task are available from the data repository of Contreras-Huerta et al (https://osf.io/qmpw7/overview?view_only=55383fa079d04acc9838d28812621f32), and the source data for the rat task are available from https://doi.org/10.7554/eLife.48429.005.

Research involving human participants, their data, or biological material

Policy information about studies with human participants or human data. See also policy information about sex, gender (identity/presentation), and sexual orientation and race, ethnicity and racism.

Reporting on sex and gender	Sex and gender-based analyses were not performed. The berry-human task considered self-reported gender in study design to obtain balanced samples, reported in the original articles. The field-human task did not include gender reporting in the original manuscript.
Reporting on race, ethnicity, or other socially relevant groupings	Data on race, ethnicity, or other socially relevant groupings were not used in this study.
Population characteristics	Field-human dataset: 40 healthy human volunteers. One participant was excluded due to poor engagement with task, final sample size of 39 (mean age 24, range 20–30 years).<br>Berry-human dataset: 31 healthy human volunteers. Two participants were excluded due to very short (<500 ms) or long (>3 s.d. from the sample average) leaving times indicating poor task understanding. The final sample size was 29 (age m=24.7, s.d.=5.0).<br>Rat dataset: Adult Long-Evans rats (Charles River, Kingston, NY).
Recruitment	Human tasks recruited volunteers through participant databases, and online platforms advertising studies to University of Oxford students and the local Oxford community.
Ethics oversight	Field-human: University of Oxford ethics committee<br>Berry-human: University of Oxford ethics committee (R55988/RE001)<br>Rat: From the original article - 'This study was performed in strict accordance with the recommendations in the Guide for the Care and Use of Laboratory Animals of the National Institutes of Health. All procedures were approved by the Princeton University (Protocol 1969) and Rutgers University (Protocol 14-075) Institutional Animal Care and Use Committees.'

Note that full information on the approval of the study protocol must also be provided in the manuscript.

Field-specific reporting

Please select the one below that is the best fit for your research. If you are not sure, read the appropriate sections before making your selection.

☐ Life sciences ☒ Behavioural & social sciences ☐ Ecological, evolutionary & environmental sciences

For a reference copy of the document with all sections, see nature.com/documents/nr-reporting-summary-flat.pdf

Behavioural & social sciences study design

All studies must disclose on these points even when the disclosure is negative.

Study description	Quantitative experimental studies examining choices in a decision-making task.
Research sample	Existing datasets were used from Le Heron et al (2020) study 1: https://doi.org/10.1523/JNEUROSCI.2586-19.2020 for the field-human task; Contreras-Huerta et al (2024) study 2: https://doi.org/10.1038/s41598-024-69452-x for the berry-human task; Kane et al (2017) travel-time experiment: https://doi.org/10.7554/eLife.48429 for the rat task.<br><br>Field-human: 40 people (aged 20-30 years, M = 24), recruited through the University of Oxford communities.<br>Berry-human: 31 people (aged M = 24.7, SD = 5), recruited through the University of Oxford communities.<br>Rat: 8 adult Long-Evans rats (Charles River, Kingston, NY).
Sampling strategy	For the human datasets, participants were recruited through the University of Oxford communities using convenience sampling. The original article for the berry-human task reports that sample size was determined based on statistical simulations from a previous

	study, which identified that a sample size between 25-30 participants was sufficient to capture the main and interaction effects from the previous study with a power > 0.8. Sample size considerations were not reported in the original articles for the field-human and rat dataset.
Data collection	Human participants completed the study on a laptop with a researcher present in the room to provide guidance if necessary.<br><br>Rat task: From the original article - 'Rats were housed on a reverse 12 hr/12 hr light/dark cycle. All behavioral testing was conducted during the dark period. Rats were food restricted to maintain a weight of 85–90% ad-lib feeding weight, and were given ad-lib access to water. '
Timing	This information was not reported in the original articles.
Data exclusions	Field-human task: One participant was excluded due to poor engagement with task, identified at debriefing.<br>Berry-human dataset: Two participants were excluded due to very short (<500 ms) or long (>3 s.d. from the sample average) leaving times indicating poor task understanding.<br>Rat task: no rats were excluded.
Non-participation	To our knowledge, no participants declined participation or withdrew from the compiled dataset. This information was not reported in the original articles.
Randomization	The study used a within-subject design that randomized the task order between participants; no random group allocation was necessary.

Reporting for specific materials, systems and methods

We require information from authors about some types of materials, experimental systems and methods used in many studies. Here, indicate whether each material, system or method listed is relevant to your study. If you are not sure if a list item applies to your research, read the appropriate section before selecting a response.

Materials & experimental systems

n/a	Involved in the study
☒	☐ Antibodies
☒	☐ Eukaryotic cell lines
☒	☐ Palaeontology and archaeology
☐	☒ Animals and other organisms
☒	☐ Clinical data
☒	☐ Dual use research of concern
☒	☐ Plants

Methods

n/a	Involved in the study
☒	☐ ChIP-seq
☒	☐ Flow cytometry
☒	☐ MRI-based neuroimaging

Animals and other research organisms

Policy information about studies involving animals; ARRIVE guidelines recommended for reporting animal research, and Sex and Gender in Research

Laboratory animals	Adult Long-Evans rats were used (Charles River, Kingston, NY). Specific age was not reported in the original article.
Wild animals	The study did not use wild animals.
Reporting on sex	This information was not reported in the original article.
Field-collected samples	The study did not use samples collected from the field.
Ethics oversight	Rat: From the original article - 'This study was performed in strict accordance with the recommendations in the Guide for the Care and Use of Laboratory Animals of the National Institutes of Health. All procedures were approved by the Princeton University (Protocol 1969) and Rutgers University (Protocol 14-075) Institutional Animal Care and Use Commit

Note that full information on the approval of the study protocol must also be provided in the manuscript.

Plants

Seed stocks	*Report on the source of all seed stocks or other plant material used. If applicable, state the seed stock centre and catalogue number. If plant specimens were collected from the field, describe the collection location, date and sampling procedures.*
Novel plant genotypes	*Describe the methods by which all novel plant genotypes were produced. This includes those generated by transgenic approaches, gene editing, chemical/radiation-based mutagenesis and hybridization. For transgenic lines, describe the transformation method, the number of independent lines analyzed and the generation upon which experiments were performed. For gene-edited lines, describe the editor used, the endogenous sequence targeted for editing, the targeting guide RNA sequence (if applicable) and how the editor was applied.*
Authentication	*Describe any authentication procedures for each seed stock used or novel genotype generated. Describe any experiments used to assess the effect of a mutation and, where applicable, how potential secondary effects (e.g. second site T-DNA insertions, mosiacism, off-target gene editing) were examined.*
